# Local Conditions of Geography and Population, as Bearing on the Water Supply and Sewerage of Buffalo1Illustrated lecture before the medical section of the Buffalo Academy of Medicine, March 10, 1903.

**Published:** 1903-07

**Authors:** A. L. Benedict

**Affiliations:** Buffalo, N. Y.; 156 W. Chippewa Street


### BUFFALO MEDICAL JOURNAL.

VOL. XLII.—LVIII. JULY, 1903. NO. 12.

### ORIGINAL COMMUNICATIONS.

### Local Conditions of Geography and Population, as Bearing on the Water Supply and Sewerage of Buffalo.[1]

By A. L. BENEDICT, M. D., Buffalo, N. Y.

CHOLERA Asiatica and typhoid are the only essentially water-borne diseases, and of these only the latter applies to our climate, though various parasites may adventitiously contaminate the water supply. Considering the welfare of Greater Buffalo, it should be remembered that practically all of the sewage of the city passes to the east of Grand Island, and that the contamination of the water becomes successively greater as we follow the course of the Niagara River, except that below the falls, the comparatively uncontaminated water of the Canadian channel is mixed with highly contaminated water of the American side.

In any general discussion of water sanitation, we must decide between thorough filtration or other treatment of sewage and filtration of the water supply. The former is the ideal method, especially for large cities, and the sewage sludge is of sufficient value to pay the cost of maintenance, though scarcely that of the original plant. Under present conditions, however, including the common law of drainage-rights, the water supply itself must be filtered. Without regard to the duty of the present municipality to Greater Buffalo, it may be interesting to note that about 1-100,000 of the water of the east channel of Niagara River consists of actual excrementitious matter from the human and animal population of Buffalo.

While typhoid bacilli have been recovered from water after 100 days, any parasite tends to die if long kept in a nonparasitic environment, and it is exceedingly doubtful if any considerable contamination of our water supply can be due to Detroit,

1. Illustrated lecture before the medical section of the Buffalo Academy of Medicine, March 10, 1903.

Cleveland, Erie, or even Dunkirk. Still, it must be admitted that Lake Erie is subject to pretty general contamination. The bulk of our typhoid, however, is due to local contamination. When the old intake in the lower harbor was used, there was considerable prevalence of typhoid, which fell off quite abruptly when this intake was permanently abandoned in 1895.

In many respects the local geography and population are favorable. Thus, along the Canadian shore, there is no brook or stream not usually closed by a sand bar, until we reach the Welland Canal, whose current is mainly from and only exceptionally into Lake Erie. The nearest stream of considerable size is the Grand River, which drains Dunnville, Brantford and other towns, having an aggregate population of probably not over 30,000, and its discharge is 35 miles distant. Aside from Buffalo Creek, which is really a small stream, originally nearly closed by a sand bar and whose apparent magnitude is due to dredging, we have no stream entering Lake Erie till we reach Smokes Creek, beyond Stony Point. This is ordinarily closed by a sand bar during dry weather, so that natural filtration occurs and there is little drainage into it, except after percolation through soil and, until the last year, the population tributary to it has been small. Between Buffalo and Stony Point there is a large swamp which prevents ordinary habitation and destines the land to use for railroads and canal dockage. Meanwhile it acts as a natural septic tank, like the swamps along the Canadian shore of the lake. With the exception of two or three small brooks, there is no further drainage into the lake on the New York shore, until we reach Eighteen-mile Creek, so-named because it is about 14 miles from Buffalo. This creek has no contamination except from rural drainage, and is subjected to spontaneous filtration at many points during the dry season. There is a good deal of summer population along both shores of the lake.

Confining our attention to Buffalo itself, we find the geographic conditions somewhat peculiar and, on the whole, rather favorable from the engineering standpoint. Beside the lake and river, the following water courses are to be considered: Buffalo Creek, the Erie Canal, the harbor above Buffalo Creek and that along the line of the Niagara River, converging with the Erie Canal opposite Squaw Island, and finally discharging into the Niagara River through a lock at the north end of Squaw Island. These watercourses might influence the water supply of the city. Below the intake and, therefore, of importance only locally or with reference to the down-stream components of Greater Buffalo, is Scajaquada Creek, which contains a good deal of minor private drainage from the country immediately east of the city line and the northeast part of the city, all of which passes through Forest Lawn Cemetery. Although there are some large springs whose flow is through the cemetery into the creek, this flow is probably not directly through graves and, without such flow, there is very little possibility of the escape of pathogenic germs from corpses. On account of its rock bottom, there is little spontaneous filtration for Scajaquada Creek, but its expansion into the Park Lake constitutes a sedimentation tank, and there must be considerable spontaneous purification by saprophytes and macroscopic, aquatic plants. Entering the river at the foot of Ontario street is Cornelius Creek, a good sized stream twenty years ago, but now almost entirely drained into sewers, except during freshets.

Between Scajaquada Creek on the north, Buffalo Creek on the south, and Bailey avenue on the east, most of the city is sewered into Niagara River at Albany street below the water intake. North Buffalo discharges into the river at Ontario street. South and southeast Buffalo discharge into Buffalo Creek and its branches, and what is termed the "Island," between Buffalo Creek and the outer harbor, is partly sewered through Ganson street into Buffalo Creek. The outer harbor, above Buffalo Creek, is almost entirely inclosed by the breakwater, which, by the way, is the most extensive in the world, and there is no drainage of any kind into it, except through a considerable distance of sand, and except the drainage into a canal of the steel plant at Stony Point. With the exceptions just noted, the Buffalo Creek, Erie Canal and harbor below Buffalo Creek, receive only a slight local drainage until Scajaquada Creek is reached, where there is an overflow from the regular sewer to allow the discharge of rain water. Below this point, there is considerable local drainage into the canal, which is bad for Tonawanda and Lockport, but which does not affect Buffalo to a material extent. The Hamburg Canal is now a dead issue, but even at its worst it was probably not a source of much pathogenic contamination on account of the enormous development of saprophytes.

At the present time, allowing for imported cases of typhoid, our typhoid incidence is not very high and our water supply is much better than that of Philadelphia, for instance, and better than that which we formerly drew from the harbor. I do not believe that we derive any considerable number of typhoid bacilli from distant cities. The United States Engineering Bureau reports that there are no appreciable currents in the lake, and the chances are very slight that a typhoid bacillus even from the Grand River or from Dunkirk should survive under the unfavorable surroundings of nonparasitic conditions, or should fail to be sedimented. The circumstances are far different so far as concerns the seven-hour trip from the Buffalo trunk sewer to Niagara Falls.

Under ordinary conditions, the breakwaters protect the emerald channel in which the intake is situated from contamination by Buffalo Creek; not that the breakwaters themselves are watertight, but that they serve to guide the currents. The real protection against contamination from shore currents on either side, is the natural force of the central current. The steel plant district and West Seneca are terribly contaminated with typhoid, and the sandy soil and ignorance of many of the residents are bound to lead to infection by dust and insects when dry weather comes. The very badness of the local condition is the main protection of Buffalo. There is practically no sewerage system at West Seneca and, instead of a direct discharge through Smokes Creek, we are liable to contamination of our water supply only from surface washing during floods and in the spring thaws.

On the other hand, during the spring freshets, the waters of Buffalo Creek may be carried out so as to contaminate the emerald channel and, according to the condition of the wind, the lake level is subject to frequent changes of five feet or more, so that shore water may be driven back and forth and may reach the intake from either shore. Then, too, during the summer, the enormous number of vessels leaving Buffalo Creek may carry bacteria into the emerald channel. Only four ports in the world have more shipping than Buffalo. Finally, it must be considered that in spite of what has been said, Lake Erie is not entirely free from typhoid contamination and is likely to become more contaminated as the population increases along its shores.

It has been suggested that the danger of contamination of our water supply may be immediately reduced, so far as Buffalo and Smokes Creek and American shore pollution are concerned, by opening the lock in the harbor at Squaw Island, so as to increase the harbor current. This suggestion is worth acting upon. So far as sewage disposal is concerned, it would be possible at considerable and yet not prohibitive expense, to collect all of the sewage of the city on Squaw Island, which would probably be large enough for a plant for separating sludge, and this could be sold for use on slowly growing crops, but it should never be used on market gardens. A more ideal plan would be to convey all of the sewage of Greater Buffalo to a line somewhere between the present north city line and Tonawanda, and to establish a sewage farm somewhere about Rosedale. At least 500 acres would be necessary. Or, to avoid the expense of tunneling Buffalo Creek, the sewage of south and southeastern Buffalo, including West Seneca and the Steel Plant, might be led to a separate farm. The fall of Buffalo Creek within the city limits is very little, barely ten feet, so that, in carrying sewage away from the lake, less pumping would be necessary than in delivering at a sewage farm to the north of the present city limits.

Without regard to sewage disposal, it will be necessary to filter the water supply, in order to secure protection from typhoid. It has been proposed to carry the water inlet farther up into the lake. This would apparently do away with the danger of contamination from Buffalo Creek, including ordinary sewage and rural drainage. The market gardens east of the city are freely fertilised and, up to a few years ago, to a considerable degree with nightsoil. At present there is not much human excrement used. But there is now practically no danger from Buffalo Creek, except for a few days in a year, from increase of current by freshets and from the rise and fall of the lake level, which is equivalent to a tide of ten feet or more between high and low water, but which is due to banking of the water by winds. This rise and fall endangers the purity of our water supply from water normally in contact with one shore or the other, and it is practically impossible to carry the inlet far enough into the lake to do away with the tidal danger, and particularly to obviate the danger from West Seneca, where typhoid is liable to be endemic for several years. Even if we secured a location free from present contamination, there is no knowing when some Canadian enterprise may change the local conditions as suddenly as has been the case at Stony Point. We must remember that the unusually favorable commercial circumstances at the eastern end of Lake Erie, promise a large population in the comparatively near future and that the slow development of the past has been due solely to an abnormal lack of local enterprise.

I believe thoroughly that we cannot hope, even by the most tremendous engineering feat, to get a source of water supply essentially better, except temporarily, than the present. The experience of European cities, dealing with a water supply much more highly contaminated than that of Lake Erie can become, without any special care as to sewage, so far as we can now foresee, is that sand filtration may be expected to reduce typhoid mortality to about 2 per 100,000 population. The actual results for several European cities vary from this up to 9:100,000. Artificial filters of various kinds have been used by several small American cities, but they do not produce as good results and, in four or five cities, the incidence of typhoid has actually increased from 20 to 70 per cent.

Buffalo is at present pumping rather more than 100 gallons of water per capita per diem. It would hardly be worth while to filter this entire amount, for the contamination is negligible for sprinkling and fire purposes and for purely commercial uses. A domestic supply of 30 gallons per capita would be ample, and Dr. Greene, the commissioner of health, informs me that an acre of sand filter can yield from 1,000,000 to 1,500,000 gallons daily. Hence, 12 acres would suffice to purify our present need of domestic water supply. So large a tract is not available near the present reservoir, and the elevation of the reservoir is only about 80 feet, while the maximum elevation of the north-eastern part of the city is from 100 to 120 feet, so that we have to rely upon Holly pressure for a considerable part of the city. The necessary land could be found near the present pumping station, either on Squaw Island or near the Front. The proposal to place a general reservoir in the Hamburg Hills, to supply the entire American Frontier, involves too great expense to be considered at present. Even by going out into the lake, at this point, it would still be necessary to establish a sand filter, but the latter could be easily and quite cheaply secured.

In conclusion, I wish to express my thanks to Dr. Greene and other members of the local health department, to Mr. Fell, of the engineering department, and to Mr. Green and Mr. Odell and, through them to the firm of Matthews & Northrup, for the loan of the interesting and complete maps which have been used as illustrations.

NOTE.—In summing up, Dr. Benedict expressed the belief that Smokes Creek could easily be turned inside the breakwater, but that the principal immediate danger is from accidental surface drainage, there being practically no sewerage system at West Seneca. While not ascribing any large proportion of present contamination to distant sources, he agreed with Dr. Hopkins and Dr. Greene that there was an increasing general contamination of the water of Lake Erie. The breakwaters did not directly protect against shore contamination, but served to guide the currents. The real protection was by the force of the currents themselves. He did not believe that the speed of floating objects could be relied upon to indicate the progress of submerged material, such as sewage particles, the latter being protected from wind. The tendency was for all such particles to be driven toward the shore. The sulphurous taste of water during the winter indicated flow from springs in the tunnel itself, and there might be contamination from the overlying harbor.

156 W. CHIPPEWA STREET.

